# OA22 Juvenile idiopathic arthritis in transition: From childhood remission to difficult-to-treat adult disease

**DOI:** 10.1093/rap/rkag113.022

**Published:** 2026-11-03

**Authors:** Krishna Dasigan, Najla Elndari, Hassan Butt, Rihards Buss

**Affiliations:** The Newcastle upon Tyne Hospitals NHS Foundation Trust, Newcastle-upon-Tyne, United Kingdom; The Newcastle upon Tyne Hospitals NHS Foundation Trust, Newcastle-upon-Tyne, United Kingdom; The Newcastle upon Tyne Hospitals NHS Foundation Trust, Newcastle-upon-Tyne, United Kingdom; The Newcastle upon Tyne Hospitals NHS Foundation Trust, The Newcastle upon Tyne Hospitals NHS Foundation Trust, United Kingdom

## Abstract

**Introduction:**

Juvenile idiopathic arthritis (JIA) frequently persists into adulthood, yet patients achieving prolonged remission during adolescence are often expected to have favourable long-term outcomes. Transition from paediatric to adult rheumatology represents a vulnerable period where disease relapse, treatment interruption and evolving management challenges may emerge. We present a patient with extended oligoarticular ANA-positive JIA diagnosed aged two years who maintained disease control for many years before developing severe, treatment-refractory polyarticular disease following transition to adult services. This case highlights the complexities of transitional care, biologic sequencing, immunogenicity and the cumulative burden of chronic glucocorticoid dependence.

**Case description:**

A male patient was diagnosed with ANA-positive extended oligoarticular JIA at two years of age after recurrent synovitis affecting the left knee, ankle, elbow and wrist. Initial management included repeated intra-articular corticosteroid injections followed by methotrexate because of persistent disease activity. Bilateral anterior uveitis developed early in the disease course and responded to topical corticosteroids.

Throughout childhood, disease activity gradually improved with methotrexate alongside physiotherapy and supportive multidisciplinary input addressing hypermobility, musculoskeletal rehabilitation and school-related difficulties. Despite intermittent knee flares requiring intra-articular corticosteroid injections, prolonged remission was achieved. Methotrexate was successfully withdrawn in 2013 before relapse of arthritis and uveitis in 2014 necessitated recommencement of therapy. Ongoing methotrexate intolerance led to adalimumab initiation in 2015 with sustained remission for over two years.

As transition approached, treatment de-escalation was considered but deferred until transfer to adult services. Following a missed adolescent follow-up during the COVID-19 pandemic, he re-presented with severe bilateral knee synovitis, inflammatory marker elevation and marked functional deterioration. Disease rapidly evolved into persistent polyarticular inflammatory arthritis involving knees, ankles, hips, wrists, elbows and hands.

Subsequent management proved challenging. Anti-adalimumab antibodies developed following biosimilar reintroduction. Sequential therapies including etanercept, infliximab, tocilizumab, abatacept, upadacitinib and certolizumab, alongside repeated methotrexate courses, failed to achieve sustained disease control. Recurrent respiratory, urinary and ENT infections repeatedly interrupted biologic therapies leading to longer periods trialling these agents with steroid use in between. Numerous intra-articular corticosteroid injections, intravenous methylprednisolone admissions and prolonged oral prednisolone dependence resulted in cushingoid features. Additional diagnostic uncertainty surrounding possible inflammatory bowel disease further complicated biologic selection. At 23 years old he remains steroid-dependent with active inflammatory polyarthritis despite multiple advanced therapies and ongoing multidisciplinary review.

**Discussion:**

This case illustrates the unpredictable long-term trajectory of JIA despite excellent disease control throughout childhood. Although extended oligoarticular JIA frequently responds well to conventional DMARDs and TNF inhibition, remission does not necessarily predict sustained disease quiescence in adult life. Following transition, this patient experienced transformation into aggressive, refractory inflammatory arthritis requiring sequential advanced therapies with persistent glucocorticoid dependence.

Several factors likely contributed to the complexity of management. Immunogenicity was demonstrated through the development of anti-adalimumab and later infliximab antibodies, limiting therapeutic efficacy. Recurrent infections repeatedly interrupted treatment, making adequate assessment of treatment response difficult and delaying steroid reduction. Potential co-existent inflammatory bowel disease further restricted therapeutic choices while prolonged glucocorticoid exposure resulted in significant treatment-related morbidity.

Importantly, this case also demonstrates that successful transition encompasses considerably more than transfer between services. During childhood, coordinated multidisciplinary care addressed musculoskeletal rehabilitation, ophthalmological surveillance, education and psychosocial challenges including school bullying. Disease relapse occurred during the transition period following a missed review, emphasising how vulnerable this stage can be for young people with chronic rheumatic disease. Whether earlier re-engagement would have altered disease trajectory cannot be determined, but maintaining continuity of specialist care remains essential.

Finally, this case highlights the increasingly complex therapeutic landscape in refractory adult JIA. Despite exposure to multiple biologic classes and targeted synthetic DMARDs, disease control remains elusive, requiring multidisciplinary discussion regarding unconventional combination therapy, alternative cytokine targets and clinical trial enrolment. Recognition of refractory JIA as a distinct phenotype requiring individualised management is becoming increasingly important as more children with JIA survive into adulthood with ongoing active disease.

**Key learning points:**

• Extended oligoarticular JIA may relapse after prolonged remission and evolve into severe refractory inflammatory arthritis during adulthood.

• Transition from paediatric to adult rheumatology is a high-risk period requiring robust continuity of care, even in patients with longstanding remission.

• Immunogenicity to TNF inhibitors, recurrent infections and treatment interruptions can significantly complicate biologic sequencing and assessment of therapeutic response.

• Long-term glucocorticoid dependenceremainsa major source of morbidity despite the availability of multiple biologic and targeted synthetic therapies.

• Management of refractory adult JIA requires close multidisciplinary collaboration between adult and paediatric rheumatology, ophthalmology, gastroenterology, physiotherapy,psychologyand specialist nursing teams.

• This case reinforces that successful transition should be viewed as a longitudinal process rather than a single transfer event, with sustained engagement andmonitoringessential to optimise long-term outcomes.

